# High-Pressure Response of Amyloid Folds

**DOI:** 10.3390/v11030202

**Published:** 2019-02-28

**Authors:** Joan Torrent, Davy Martin, Angélique Igel-Egalon, Vincent Béringue, Human Rezaei

**Affiliations:** 1 MMDN, Univ. Montpellier, EPHE, INSERM, U1198, F-34095 Montpellier, France; 2 Institut National de la Recherche Agronomique, UR892, Virologie Immunologie Moléculaires, F-78350 Jouy-en-Josas, France; davy.martin@inra.fr (D.M.); angelique.egalon@inra.fr (A.I.-E.); vincent.beringue@inra.fr (V.B.); human.rezaei@inra.fr (H.R.)

**Keywords:** amyloid, high pressure, prion, polymorphism

## Abstract

The abnormal protein aggregates in progressive neurodegenerative disorders, such as Alzheimer’s, Parkinson’s and prion diseases, adopt a generic structural form called amyloid fibrils. The precise amyloid fold can differ between patients and these differences are related to distinct neuropathological phenotypes of the diseases. A key focus in current research is the molecular mechanism governing such structural diversity, known as amyloid polymorphism. In this review, we focus on our recent work on recombinant prion protein (recPrP) and the use of pressure as a variable for perturbing protein structure. We suggest that the amyloid polymorphism is based on volumetric features. Accordingly, pressure is the thermodynamic parameter that fits best to exploit volume differences within the states of a chemical reaction, since it shifts the equilibrium constant to the state that has the smaller volume. In this context, there are analogies with the process of correct protein folding, the high pressure-induced effects of which have been studied for more than a century and which provides a valuable source of inspiration. We present a short overview of this background and review our recent results regarding the folding, misfolding, and aggregation-disaggregation of recPrP under pressure. We present preliminary experiments aimed at identifying how prion protein fibril diversity is related to the quaternary structure by using pressure and varying protein sequences. Finally, we consider outstanding questions and testable mechanistic hypotheses regarding the multiplicity of states in the amyloid fold.

## 1. Introduction

A polypeptide chain can be involved in several misfolding pathways, each producing a plethora of structurally distinct multimeric assemblies [1]. Amyloidogenesis is considered a critical pathway to be investigated since it generates oligomers and amyloid fibrils [2]. These proteinaceous assemblies are thought to be responsible for the cellular toxicity and the autocatalytic self-propagation of the aberrant fold, leading to the pathogenesis of fatal neurodegenerative disorders such as Alzheimer’s and prion diseases [3]. The probable cause of these diseases is now collectively referred to as propagated protein misfolding, and refers to the process by which an amyloid fold is propagated throughout the brain by inducing normally folded proteins to misfold into self-assemblies of the same conformation. However, the precise conformation adopted by the amyloid fold is not fully understood. This lack of understanding is partly driven by the existence of multiple-related amyloid conformations known as amyloid fibril polymorphism [4]. These various amyloid forms are thought to be mechanistically related to prion strains in mammals. The prion protein (PrP) involved in the pathogenesis of prion disease is known to adopt multiple pathological conformers. These unique conformers or strains can be fully transmitted and underlie distinct prion disease phenotypes [5,6,7]. Hence, the concept of amyloid strains goes beyond the mammalian prion field and includes all neurodegenerative disorders with a prion-like mechanism of propagation [8].

Given the present view of protein folding, oligomers and amyloid fibrils are expected to occupy various minima within a region of the funnel energy landscape that is dominated by intermolecular contacts. Oligomers are formed transiently and evolve to higher order assemblies. Conversely, fibrils are thermodynamically stable and are often considered to be the energetically most favorable protein state [9], in accordance with their known tissue persistence. Structural studies have shown that amyloid fibrils generated in vitro, from purified recombinant proteins or synthetic peptides, are composed of a cross-β structure, in which the protomers are stably assembled by repetitive hydrogen-bonding that extends the length of the fibrils. From a structural point of view, amyloid fibril polymorphism is a result of an altered cross-β organization, and involves variations in the lengths of the fibril spine and connecting loops, location of the folds, as well as differences in the arrangement of β-strands [10]. These structural reorganizations undeniably affect the thermodynamic stability as well as the compactness of the amyloid fibril structure. However, detailed molecular insights into the internal packing environments of the amyloid fold still remain elusive. Therefore, we recently used protein denaturation approaches (using a chaotropic agent) to study the diversity of the infectious prion quaternary structure [11]. We revealed that the strain structural determinant is enciphered in the packing of a highly stable oligomeric subunit constituting the elementary PrP building block. However, our understanding of the hydration and packing arrangement within the prion core region, which directly affect the volumetric properties of such assemblies, is still rather limited. Thus, we need complementary approaches in order to provide such novel insights.

The ability to make in vitro fibril amyloids from purified recombinant PrP (recPrP) provides a model system where the structural/functional properties of these PrP assemblies can be elucidated. In addition, the opportunity to pressurize/depressurize biological molecules at will provides an approach to studying these features [12]. The effects induced by high-pressure on protein folding and aggregation have been covered in a number of reviews. The details of how high-pressure experiments affect thermodynamics, kinetics and structure is beyond the scope of this review. For a fuller discussion, see [13,14,15,16,17]. As a supplement, we here briefly describe the structural origins of pressure unfolding of proteins as well as the strong pressure dependence of the structural transformations that we have previously reported for recPrP.

An increase/decrease in pressure propagates rapidly and maintains the solvent properties. The use of pressure allows the dissection of the conformational state of a protein in terms of its volumetric properties. Under very high pressures (1–6 kbar or 100–600 MPa), voids within a protein folded structure become unstable and cause the protein to unfold [18]. The contribution to the change in free energy due to pressure is given as pΔ*V*. The change in partial molar volume upon unfolding, Δ*V*, is typically negative, making the free-energy of the unfolded state lower than the folded state. In analogy with the process of protein folding/unfolding, the high pressure-induced effects of which have been studied for more than a hundred years, several recent studies have also shown that the amyloid fold is perturbed under pressure [19,20,21,22,23,24]. Since the dissociation reaction is favored under pressure, the volume change of the system must be negative (i.e., amyloid fibrils present cavities). By assuming that amyloid barostability reflects the system volume changes accompanying amyloid dissociation under pressure, and that different amyloid folds have different degrees of compactness, the structural and dynamic response of an amyloid to pressure can vary significantly depending on its conformational properties [24]. Spectroscopic properties that can be measured under pressure (e.g., fluorescence, absorption, FTIR, Raman, and NMR) provide details of the underlying conformational changes.

The experimental results reviewed here, as well as preliminary data obtained using fibrils generated from different species-specific recPrP proteins, allow us to discuss whether multiple stable and conformationally distinct PrP assemblies can be distinguished by their volumetric properties.

## 2. Sensitivity to Pressure of the α-Helical recPrP Fold

We showed that the α-helical form of recPrP (α-recPrP) partially unfolds under high pressure (up to 600 MPa) [25]. The pressure-induced reaction followed a simple two-state and reversible transition leading to a metastable structure, possibly a soluble oligomer with an enhanced thioflavin T (ThT) binding capacity. The solubility of the PrP conformer populated at high pressure was reduced by increasing the protein concentration and pH. At pressures above 400 MPa (pH 8.5), we obtained an irreversible aggregation process that was accompanied by an increase in the fluorescence intensities of 8-anilino-1-naphthalenesulfonic acid (ANS) and ThT, indicating that the intermediates involved in the aggregation process adopted a partially unfolded and amyloidogenic conformation. PrP aggregates were formed after prolonged incubation under high pressure and showed a typical amyloid fibril structure when observed by transmission electron microscopy. These fibrils, which coexisted with non-amyloid aggregates, were capable of ThT and Congo red binding. In addition, they showed a strong β-sheet content and were resistant to PK digestion. However, unlike PrP fibrils formed using chemical denaturants [26], they exhibited no ANS binding ability. This result was consistent with the occlusion of exposed hydrophobic regions due to the unique favorable intermolecular contacts established under high pressure. Our finding that applying high pressure to α-recPrP induces an alternative misfolding pathway accompanied by a decrease in volume and leading to a structurally distinct amyloid is conceptually attractive and might be used to provide mechanistic insights into the etiology of PrP polymorphism.

## 3. Sensitivity to Pressure of the β-Sheet-Rich recPrP Assemblies

We then asked whether the quaternary, tertiary, and secondary structural features of preformed β-sheet-rich recPrP assemblies were affected by pressure. We showed that upon pressure perturbation, PrP oligomers and amyloid fibrils dissociate into monomeric PrP molecules that refold into the native α-helical conformation after pressure release [20,24]. In addition, we asked whether pressure sensitivity was dependent on the amyloid morphotype.

### 3.1. Oligomers

To do so, we initially focused our work on the characterization of the kinetic and energetic behavior under pressure of misfolded recPrP oligomers of different sizes [24]. Different stable and non-fibrillar β-sheet-rich oligomers formed from α-recPrP, named O0 (~82 mer), O1 (~36 mer) and O3 (~12 mer), were generated upon heating and purified from the heterogeneous mixture of PrP conformers. We showed that the different PrP oligomers had distinct pressure-sensitivities. The barostability was O3 > O0 > O1. We concluded that high molecular weight complexes, which are more prone to be enriched in void volumes in the intermolecular boundaries, are preferentially destabilized by pressure due to the larger volume decrease upon dissociation, compared with smaller oligomers. The apparent discrepancy found between the response of O0 and O1 to high pressure was explained by the fact that the homoassociation of O1, which leads to the population of O0 [27], could encompass conformational rearrangements that collapse potential packing defects or even create favorable interface interactions. We then focused on their kinetic and energetic behavior under pressure. The biphasic kinetics obtained for O1 were readily explained by the transient formation of a kinetic intermediate. Nevertheless, we recently revealed the existence of at least two structurally distinct sets of assemblies, termed Oa and Ob, forming the O1 oligomer [28]. Therefore, a plausible explanation for the biphasic relaxation profiles observed is that we reported on both subpopulations. We also showed a large negative activation volume for the dissociation reaction, together with an increase in both apparent activation enthalpy and entropy, and suggested a transition state ensemble that is less structured and significantly more hydrated than the oligomeric state. We concluded that defects in atomic packing may deserve consideration as a new factor that influences the PrP assembly puzzle.

### 3.2. Amyloid Fibrils

With the results of the oligomer-type specific effect observed under pressure, and the results from the previous literature underlining the importance of the PrP amino acid sequence in both prion strain and transmission barrier phenomena [29], we sought to characterize the barostability properties of species-specific recPrP fibrils to gain insight into the fibril packing.

Our recent results obtained by applying sedimentation velocity ultracentrifugation to a panel of prion strains were consistent with the essential role of the hydrodynamic properties (hydrodynamic size) of PrP^Sc^ in prion conversion efficiency and the duration of disease [30]. Another key parameter distinguishing various PrP^Sc^ superstructures and differing between PrP^Sc^ and PrP^C^ is the density [31]. Of particular interest is that PrP^Sc^ was found in fractions of lower density than PrP^C^ after isopycnic density gradient centrifugation (i.e., equilibrium separation). This observation is consistent with the presence of packing defects that might arise during the assembly of PrP protomers to form PrP^Sc^ assemblies. In line with these findings, using mature amyloid PrP fibrils we demonstrated that the dissociation reaction induced by pressure leads to a lower volume monomeric PrP state. We showed a nearly complete and irreversible loss of ThT binding to the fibrils treated under high pressure. As for PrP oligomers, the kinetic rates were strongly pressure and temperature dependent, and the reaction occurred via a mechanistically similar unfolding process that implied a disordered and hydrated kinetic transition state.

**Figure 1:**
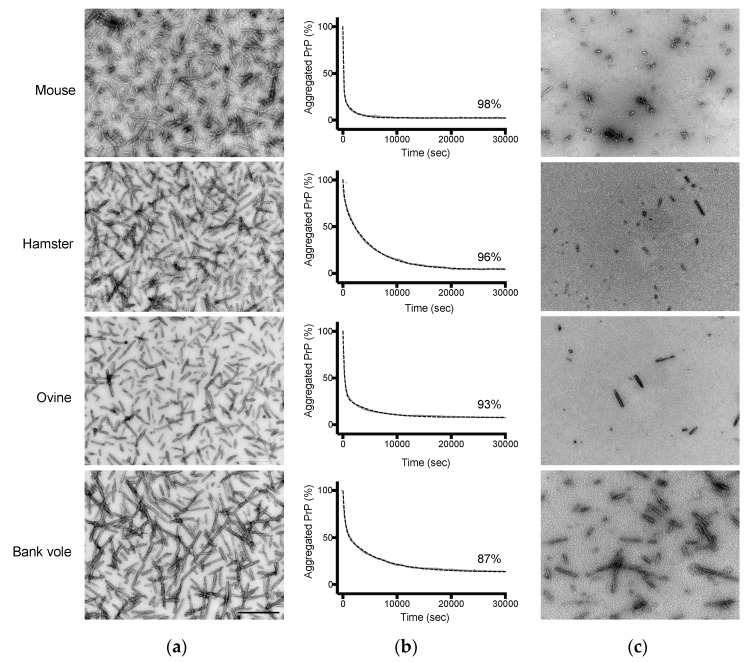
Pressure dissociates PrP fibrils and alters their macrostructure. Negative stained transmission electron microscopy micrographs of (**a**) untreated (8 h at 25 °C) PrP fibrils; and (**c**) after a cycle of compression (360 MPa, 8 h)/decompression at 25 °C. Scale bar, 500 nm. (**b**) The kinetics of the pressure-induced PrP fibril dissociation were recorded as a decrease in light scattering. Fibril fraction was calculated from the amount of resolubilized PrP rescued after the pressure treatment, as judged from the absorbance at 280 nm of the supernatants obtained after removing fibrils by centrifugation. Dashed lines, linear fits to the data.

Based on these initial studies, we formed amyloid fibrils from recPrP of mouse, Syrian hamster, ovine (genotype ARQ/ARQ), and bank vole. Transmission electron microscopy (TEM) confirmed their presence in all PrP samples, as seen in Figure 1a, by showing an extensive array of elongated fibers. The effect of pressure on these assemblies was assessed by applying a fast increase (i.e., within 30 s) of pressure of 360 MPa at 25 °C. The kinetic profiles of the structural changes were measured by monitoring light scattering, as seen in in Figure 1b. The observed loss in scattering intensity upon pressure treatment was found to be highly reproducible, even when performing the same assay independently at different protein concentrations as seen in Figure S1 and varied depending on the fibril type.

**Figure 2:**
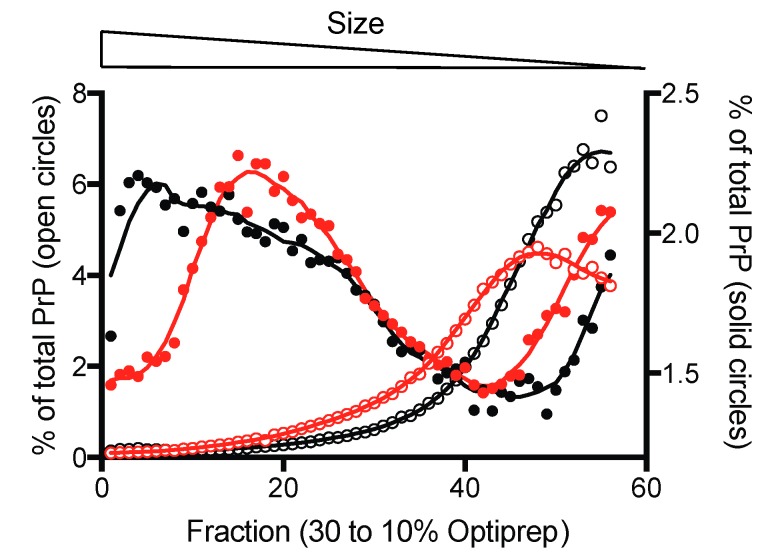
Pressure alters the size-distribution of PrP fibrils. Sedimentation velocity centrifugation in density gradients of mouse (black) and bank vole (red) PrP fibrils labelled with Alexa Fluor 488. The sedimentogram of non-treated fibrils is denoted in solid circles (right Y-axis) and those obtained after a cycle of compression (360 MPa)/decompression at 25 °C are shown in open circles (right Y-axis).

Counterintuitively, while hamster and ovine PrP fibrils exhibited comparable spectral properties using the extrinsic fluorescent dyes ThT and ANS, as seen in Figure S2, their pressure sensitivity appeared to be distinct. After decompression to atmospheric pressure, negative stain transmission electron microscopy confirmed the remarkable difference in the aggregation of the pressurized samples compared with untreated amyloid fibrils, as seen in Figure 1c, consistent with the observed decrease in scattering intensity. We next characterized the size distribution of the most contrasting recPrP fibrils (i.e., from mouse and bank vole PrP) using sedimentation velocity ultracentrifugation. Native Optiprep density gradient analysis of the PrP amyloid fibrils treated by pressure or non-treated, as seen in Figure 2, showed a clear shift in relative aggregate size after pressurization. The bulk of the fibril material shifted from the heavier to lighter fractions after a cycle of compression and decompression, indicating a reduction in the size distribution of the PrP particles. In addition, the differences in the amount of dissociated protein attained under high pressure between mouse and bank vole fibrils were further substantiated by an altered height and position of the corresponding sedimentation peak.

Because of the small sample size, our results suggesting a fibril-type-specific sensitivity to pressure dissociation have to be considered with caution. Indeed, a systematic approach using fibrils formed from a large panel of PrP variants should allow us to go beyond this proof of concept, and establish relationships between the fibril pressure response and other physicochemical properties of amyloid (i.e., ThT binding or PK-resistance). In addition, we expect that the in vitro propagation efficiency will be strongly influenced by the highly specific quaternary structural features and thus by the volume (i.e., packing) differences among the fibrils involved.

## 4. Outstanding Questions

We concluded that pressure provides an efficient tool to ascertain the highly diversified spatial arrangement between PrP protomers in an amyloid assembly. Indeed, conformational differences among PrP assemblies (i.e., oligomers or fibrils) translate into changes in the dissociation reaction attained under high-pressure (i.e., barostability). This finding allows us to hypothesize that volumetric features might underlie the amyloid polymorphism. This is crucial to gain insight into the PrP structural changes that accompany the assembly process. However, many questions remain, the resolution of which are critical to more completely understand the amyloid fold. We here outline a few of these questions. Our results raise the issue of whether such “packing” polymorphism is limited to PrP fibrils, or whether it is a common phenomenon among amyloid proteins. Related but not identical to this question, is whether the high-pressure approach could be applied to brain derived aggregates (i.e., preparations of amyloids from mammalian brain) in order to make meaningful progress towards the understanding of the etiology and progression of non-PrP neurodegenerative diseases, such as Alzheimer’s disease and its atypical presentations. Finally, the last question concerns whether the adaptation and preferential selection of minor amyloid conformations that pre-exist in the sample might be achieved under high pressure.

**Figure 3:**
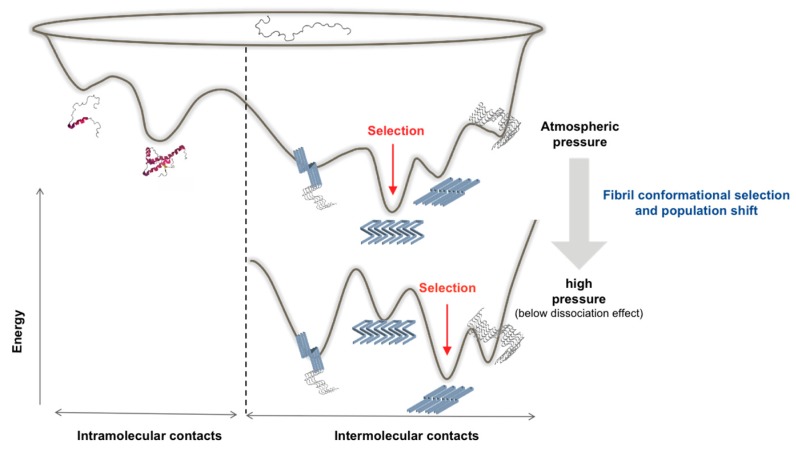
Schematic representation of a hypothetical effect of pressure on the energy landscape of an amyloidogenic protein. The conformational selection phenomenon assumes that the new thermodynamic condition reached under pressure will preferentially stabilize the amyloid state (i.e., conformation) with the highest packing density, resulting in a population shift of the ensemble toward the state showing the deepest energy minimum.

It is expected from a thermodynamic point of view that individual-specific conditions within a mammalian species will predictably lead to a dominant fibril morphotype being preferentially stabilized and propagated, in equilibrium with less populated (i.e., rare) and conformationally different fibrils and transient intermediates. In other individuals, an alternative fibril conformation can emerge via a conformational selection and population shift of the protein ensemble towards this minor subpopulation, which in this new condition presents the deepest minimum of its free energy. It is therefore reasonable to think that the amyloid sample can be forced to adapt in vitro to a new thermodynamic condition, such as at pressures below those used to completely dissociate the amyloid or unfold the protein, and thus select an amyloid state with distinct biochemical and structural characteristics (with higher degree of compactness), as seen in Figure 3. Importantly, this conformation should remain resistant to pressure treatment in subsequent passages. We therefore expect that the conformational selection under pressure could mimic the phenomenon of inter-species transmission (i.e., passage into another host that would be unable to propagate the dominant species), and provide new information on the preferential selection of minor subpopulations (i.e., strain shift or strain mutation) [32], as well as to the extraordinary phenotypic variability of Alzheimer’s disease [33].

## 5. Materials and Methods

Recombinant PrPs—Full-length PrPs were produced in *Escherichia coli* and purified as described previously [27]. Purified PrP monomers were stored lyophilized and recovered by elution through a G25 desalting column (GE Healthcare, Orsay, France). The final protein concentration was measured by optical density at 280 nm.

Formation of Amyloid Fibrils—PrP amyloid fibrils were formed using the manual setup protocol described previously in [34]. Fibril formation was monitored using a ThT binding assay [34]. Samples were dialyzed in 10 mM sodium acetate with pH 5.0. Then fibrils were collected by ultracentrifugation and resuspended in 10 mM sodium acetate with pH 5.0. A washing step was performed by repeating the ultracentrifugation and resuspension steps. All concentrations given for fibrillar PrP refer to the respective equivalent monomer concentration.

High Pressure Treatment—PrP fibrils were diluted in the same buffer to a final protein concentration of 10 μM. A piston compressed the water. Water transmitted the pressure to a 500 μL cylindrical quartz cuvette (placed directly into the optical high-pressure cell). The cuvette was sealed with a polyethylene stretch film, which was maintained by a rubber O-ring. The film separated the internal protein solution from the pressurizing liquid, and allowed for the transmission of pressure to the solution contained in the cuvette [35]. Pressure jumps consisted of rapid (i.e., 30 s) changes of the atmospheric pressure to obtain a final pressure of 360 MPa. A pressurization cycle at 360 MPa was performed by maintaining the pressure for approximatively 8 h at 25°C and by decompression of the sample to atmospheric pressure.

Light Scattering Measurements under High Pressure—Light scattering measurements were carried out using an Aminco Bowman Series 2 fluorescence-spectrophotometer (SLM Aminco) modified to accommodate a thermostatic high-pressure optical cell. Protein disaggregation was followed by monitoring the changes in scattered light at 300 nm (4 nm slit) and excited at 300 nm (4 nm slit).

Estimation of the Kinetic Parameters—The relaxation profiles of the fibril structural reactions were fitted to double exponential decays, according to Equation (1):

(1) $$I\left(t\right)=I_{0}+A\left(1-e^{-k_{\mathrm{obs}\left(1\right)}t}\right)+B\left(1-e^{-k_{\mathrm{obs}\left(2\right)}t}\right)$$

where *I*(*t*) and *I*_0_ are the fluorescence intensities at time *t* and at time 0, *A* and *B* are the phase amplitudes, and *k*_obs_ is the measured apparent rate constant at the final pressure *P*.

Transmission Electron Microscopy (TEM)—Samples were deposited on Formvar carbon-coated grids, negatively stained with freshly filtered 2% uranyl acetate, dried and viewed using a JEOL 1200EX2 electron microscope (JEOL USA, Inc, Peabody, USA).

Fluorescence Measurements— Fluorescence measurements were performed at 20°C using a FP-6200 fluorimeter (Jasco France, Bouguenais, France). Aliquots of each protein sample were incubated with 10 μM thioflavin T (ThT) for 1 min or 50 μM 8-anilino-1-naphthalene sulfonate (ANS) at room temperature for 10 min before fluorescence measurements. For ANS spectra, excitation was at 385 nm. ThT emission spectra were recorded after excitation at 450 nm. The excitation and emission slit widths were 4 nm.

Fluorescent Labelling of Fibrils—recPrP fibrils were labelled by the addition of an equimolar amount of the aminoreactive fluorescent dye Alexa Fluor 488 carboxylic acid, succinimidyl ester (Invitrogen). Labelling was performed following the manufacturer’s recommendations. Unreacted dye was removed by three cycles of sedimentation and suspension of the fibrils.

Sedimentation Velocity—Sedimentation velocity experiments were performed by carefully loading of the recPrP sample on a 4.8 ml continuous 10–30% iodixanol gradient (Optiprep, Sigma-Aldrich), with a final concentration of 10 μM. The gradients were centrifuged at 285,000 g for 45 min in a swinging-bucket SW-55 rotor. The collected fractions were analyzed by following the Alexa Fluor 488 fluorescence using a microplate reader (SAFAS Xenius XML).

## Supplementary Materials

The following are available online at https://www.mdpi.com/1999-4915/11/3/202/s1, Figure S1: High reproducibility of the pressure-induced kinetic profiles upon assaying two independent mouse PrP fibril preparations at 10 μM (light grey) and 45 μM (red). The structural change kinetics for PrP WT were recorded as a decrease in light scattering. Fibril fraction was calculated from the amount of resolubilized PrP rescued after the pressure treatment, as judged from the absorbance at 280 nm of the supernatants obtained after removing fibrils by centrifugation, Figure S2: Fluorescence emission spectra of ThT (A) and ANS (B) in the absence of protein (black line), and in the presence of hamster (blue line) and ovine (red line) PrP fibrils. a.u., arbitrary units.
