# Supplementary material for: High-Pressure Response of Amyloid Folds
Source: Viruses. 2019 Feb 28;11(3):202. doi: 10.3390/v11030202 (PMC6466028; doi:10.3390/v11030202)
Supplement: Supplementary file 1 [file viruses-11-00202-s001.pdf]

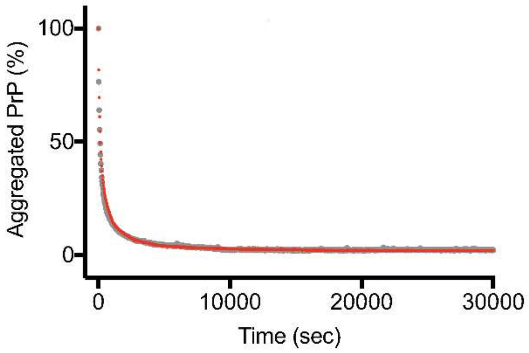

Figure S1. High reproducibility of the pressure-induced kinetic profiles upon assaying two independent mouse PrP fibril preparations at 10 μM (light grey) and 45 μM (red). The structural change kinetics for PrP WT were recorded as a decrease in light scattering. Fibril fraction was calculated from the amount of resolubilized PrP rescued after the pressure treatment, as judged from the absorbance at 280 nm of the supernatants obtained after removing fibrils by centrifugation.

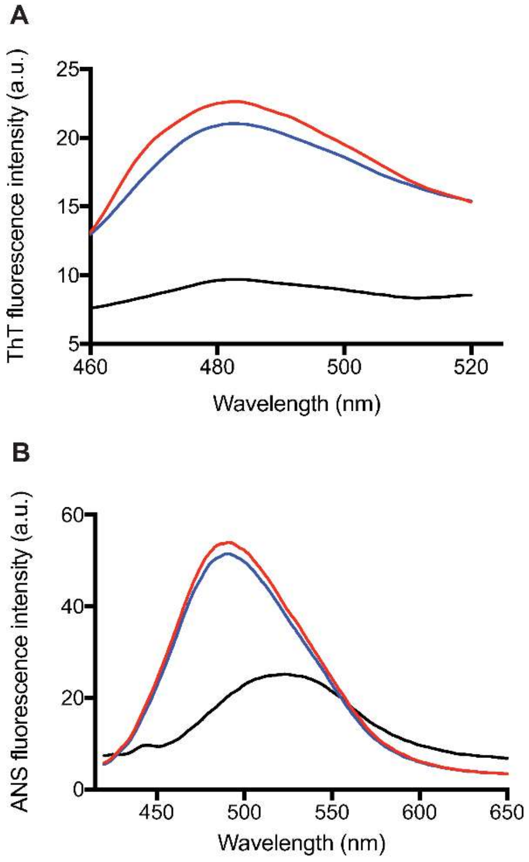

Figure S2. Fluorescence emission spectra of ThT (**A**) and ANS (**B**) in the absence of protein (black line), and in the presence of hamster (blue line) and ovine (red line) PrP fibrils. a.u., arbitrary units.
